# *Phragmites australis* +* Typha latifolia* Community Enhanced the Enrichment of Nitrogen and Phosphorus in the Soil of Qin Lake Wetland

**DOI:** 10.1155/2017/8539093

**Published:** 2017-02-19

**Authors:** Zhiwei Ge, Ran An, Shuiyuan Fang, Pengpeng Lin, Chuan Li, Jianhui Xue, Shuiqiang Yu

**Affiliations:** ^1^Co-Innovation Center for Sustainable Forestry in Southern China, College of Biology and the Environment, Nanjing Forestry University, Nanjing 210037, China; ^2^Department of Veterinary and Biomedical Science, College of Veterinary Medicine, University of Minnesota-Twin Cities, Saint Paul, MN 55108, USA; ^3^Department of Mathematics, Statistics & Computer Science, College of Science, Technology, Engineering, Mathematics and Management, University of Wisconsin Stout, Menomonie, WI 54751, USA

## Abstract

Aquatic plants play an essential role and are effective in mitigating lake eutrophication by forming complex plant-soil system and retaining total nitrogen (TN) and phosphorus (TP) in soils to ultimately reduce their quantities in aquatic systems. Two main vegetation types (*Phragmites australis* community and* P. australis* +* Typha latifolia* community) of Qin Lake wetland were sampled in this study for the analysis of TN and TP contents and reserves in the wetland soils. The results showed that (1) the consumption effect of Qin Lake wetland on soluble N was much more significant than on soluble P. (2) The efficiency of TN enrichment in wetland soil was enhanced by vegetation covering of* P. australis* and* T. latifolia*. (3) Wetland soil P was consumed by* P. australis *community and this pattern was relieved with the introduction of* T. latifolia*. (4) According to the grey relativity analysis, the most intensive interaction between plants and soil occurred in summer. In addition, the exchange of N in soil-vegetation system primarily occurred in the 0–15 cm soil layer. Our results indicated that vegetation covering was essential to the enrichment of TN and TP, referring to the biology-related fixation in the wetland soil.

## 1. Introduction

Eutrophication has now been an increasing problem in many countries. The Water Wheel reports in its recent issue that 54% of the lakes/reservoirs in Asia are impaired by eutrophication [1]. Comparatively, the percentage reaches 66% in China [2]. Total nitrogen (TN) and total phosphorus (TP) are the two major plant nutrients involved in the process of eutrophication [3, 4]. Extensive studies at the site scale have indicated that aquatic plants are effective in mitigating lake eutrophication, and many initiatives have been undertaken to improve water quality, such as introducing aquatic plants in the ecosystem and restoring or even creating wetland for this particular purpose [5]. The aquatic plants are able to form complex system with soils, where TN, TP, and some heavy metal elements cycle through various processes, such as litter decomposition, root absorption, and acid excretion, and ultimately retain N and P in soils to reduce their quantities in aquatic systems [6]. While various aquatic plants species have been adopted to treat eutrophication [7],* Phragmites australis* and* Typha latifolia* are the most commonly used aquatic plants in wetlands for the enhancement of water quality [8]. They have relatively high nutrients enrichment efficiencies compared to other plants, such as yellow flag (*Iris pseudacorus* L.) [7], contributing to effective nutrients removal from water in wetlands. However, their enrichment processes and the mechanisms are still unclear.

*P*.* australis* substantially improves the TN and TP removal efficiency in wetland ecosystem, due to its high growth rate and great capacity for nutrient accumulation in its stem, roots, and rhizomes [8]. It has a shallow-root system that often results in the nutrients enrichment occurring in surface soils through roots and rhizomes absorptions [9–11]. In addition,* P*.* australis* root biomass showed a positive correlation with the N content in aquatic system [12]. Add a few sentences of describing* P*.* australis* and* T*.* latifolia* and their abilities to enrich nutrients and their functions/mechanisms to remove N and P from water. By contrast, the enrichment efficiency of P is higher than that of N in* T*.* latifolia* [13]. It has a highly developed fine root system by which it can absorb the TN and TP in deep soil solutions and effectively increases the nutrient retention efficiency in wetland ecosystem [14]. Interestingly,* P*.* australis* and* T*.* latifolia* exhibit different tolerance to TN and TP deficiency, where* P*.* australis* is more tolerant to P limitation, while* T*.* latifolia* to N limitation [15, 16]. While a fair amount is known about the TN and TP retention efficiencies of* P*.* australis* and* T*.* latifolia* community, the nutrient retention efficiency of the mixed communities, such as* P*.* australis* and* T*.* latifolia*, is still unknown.

Qin Lake National Wetland Park is the second national wetland park in China approved by the State Forestry Administration [17]. In recent years, the water quality of the Qin Lake Wetland is dramatically decreasing due to the wastewater discharge, various pollutions, and dredged material disposal. The water N and P content has been increased by 4- and 17-fold, respectively, since the year from 1989 to 2010, which resulted in severe eutrophication [18]. Understanding the roles of the various aquatic plants in Qin Lake wetland, as well as their mechanisms by which they retain nutrients and purify water, provides theoretical support to the wetland management and the policy-making process. Here we select two typical riverfront communities (*P*.* australis* community,* P*.* australis* +* T*.* latifolia* community) in Qin Lake wetland to study the TN and TP stoichiometry in plant organs and the total N and P content in wetland soils and waters, as well as their seasonal variations, and to clarify how the two species differ in their contribution to wetland TN and TP enrichment, in order to improve understanding of the soil-vegetation nutrient cycle in wetland ecosystem and help aquatic plants management for Qin Lake.

## 2. Materials and Methods

### 2.1. Study Site

Qin Lake National Wetland Park is located in the middle of Jiangsu Province, Taizhou City, China (120°5′29.90′′E~120°6′14.70′′E, 32°37′2.70′′N~32°37′33.70′′N), 1.4 km in width (east-west) and 1.5 km in length (south-north), with an area of about 233.3 ha around. It has a humid subtropical climate with mild temperature and four distinct seasons.

Mean annual temperature is 16°C, respectively, 3.3°C in winter and 26.2°C in summer. Mean annual precipitation and relative humidity are 1031.8 mm and 80%. The average frost-free season is 220 days per year, and the annual leading wind directions are southeast wind. Present vegetation consists mainly of* P*.* australis*,* T*.* latifolia*, and so on.* P*.* australis* and* T*.* latifolia* community are the dominant plants, widely distributed in this area.

### 2.2. Experimental Design and Field Sampling

Two undisturbed sampling sites (A:* P. australis* dominated site and B:* P. australis* +* T. latifolia *dominated site) within the Qin Lake wetland were selected. 20 × 20 m control plot was established in both sites, where all the plants were removed once a month to prevent plant growth. Nine well-grown* P*.* australis* individuals were randomly collected in the two sites (outside the control plots) twice a season during February 2012–February 2013. Meanwhile, three additional plots (1 × 1 m) were selected in each site for harvesting the aboveground plant biomass at the end of growing season (late October), where plant stems, leaves, and spikes were collected separately in paper bags for analysing of plant biomass allocation. Roots were excavated and kept in polyethylene zip-top bags after removing dead roots and washing at the sampling sites. All plant materials were labeled and sent to lab for further analysis. Soil samples were collected from 0–15 cm, 15–30 cm, 30–45 cm, and 45–60 cm layers using multipoint mixing method. Flowing-water samples were collected at the locations 200 m up- and downstream of the channel in both sites. A transect was set up at each location and divided equally into 6 sections. Water sample at the 20 cm below the flow surface was collected for each section, separately kept in polyethylene bottle on ice, sealed tightly, and sent for lab analysis.

### 2.3. Determination of TN and TP in Plants, Soil, and Water

All plant samples were divided into roots, stems, leaves, and spikes, oven dried at 105°C for 15 min, and followed by 65°C for 24 hours to constant weight to measure the biomass. Dried plant samples were crushed, screened to a maximum particle size of 0.25 mm, and digested in H_2_SO_4_-H_2_O_2_ solution. TN was measured using the kjeldahl analysis methods and TP by the colorimetric molybdenum blue methods [19, 20]. Plant TN or TP storage (g/m^2^) was estimated by multiplying the biomass of each plant organs (g/m^2^) by total N or total P content (g/kg). Soil samples were air dried at room temperature, ground, and screened to a maximum particle size of 0.15 mm. TN and TP contents were measured, respectively, by the kjeldahl analysis methods and the colorimetric molybdenum blue methods. TN and TP of water samples were determined by ultraviolet spectrophotometric methods and spectrophotometric molybdate methods [21].

### 2.4. Data Analysis

TN and TP Concentrations were presented as mean values of at least three replicates. One-way analysis of variance (ANOVA) was performed using SPSS 19.0, and multiple comparisons were made by Dunnett's tests at a significant level of 0.05.

Since* P*.* australis* is the dominant species in Qin Lake wetland, a Grey Correlation Analysis was performed to study the relationship between TN and TP contents in the* P*.* australis* organs and in the soil [22].

## 3. Result

### 3.1. The N and P Contents in Upstream and Downstream Waters

TN and TP contents were both low in the downstream water relative to the upstream water (*P *< 0.05, Table 1). The reduction rate of TN content (37.3%) in the downstream water was significantly lower than that of P content (27.3%).

### 3.2. TN and TP Contents in Soils

**Figure 1:**
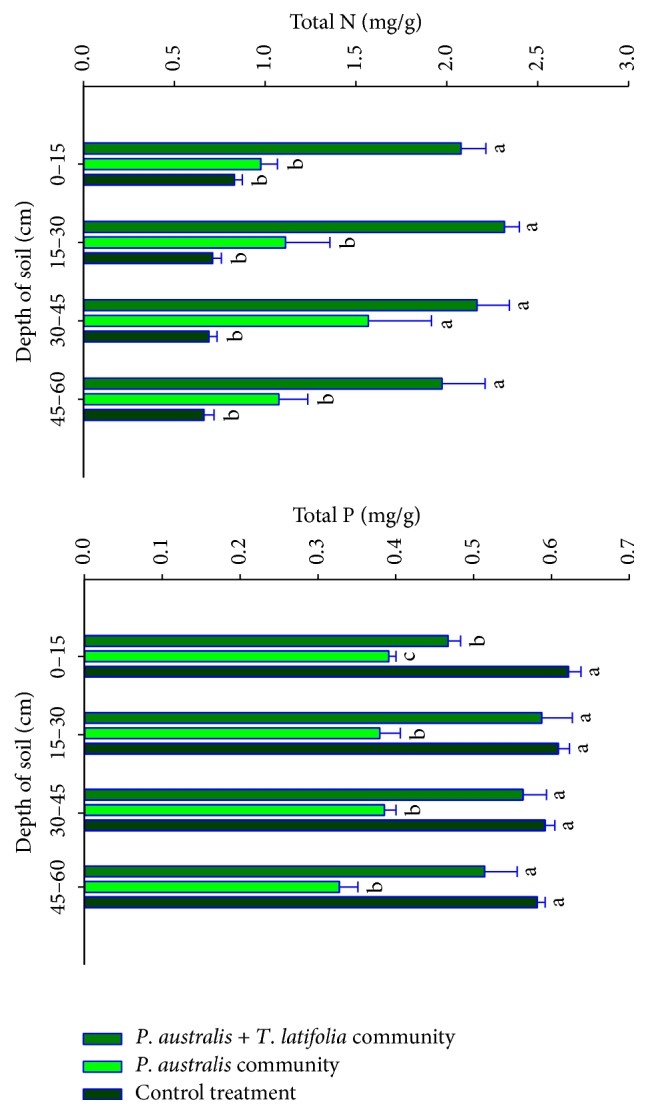
Concentrations of TN and TP in different depths of soil in experimental site. Different lowercase letters indicate significant difference among TN and TP contents of soil (*P* < 0.05). Values are mean + 1 sem.

In contrast to* P*.* australis* community,* P*.* australis* +* T*.* latifolia *community was higher in TN content in all soil layers (*P* < 0.05), while the TN content of* P*.* australis* community was higher than that of the control sites with plants harvested (0.05 <* P* < 0.08, Figure 1). The soil total P content was lowest in* P*.* australis* community (*P* < 0.01). No significant difference in total P content was observed between the soils in the mixed communities and the control sites (*P* > 0.05), except the 0–15 cm layer showing a significantly low total P content compared to the control site (*P* < 0.01). However, the TP content of* P*.* australis* community was significant lower than that of the control sites (*P* < 0.01).

### 3.3. TN and TP Contents in Plant Organs

**Figure 2:**
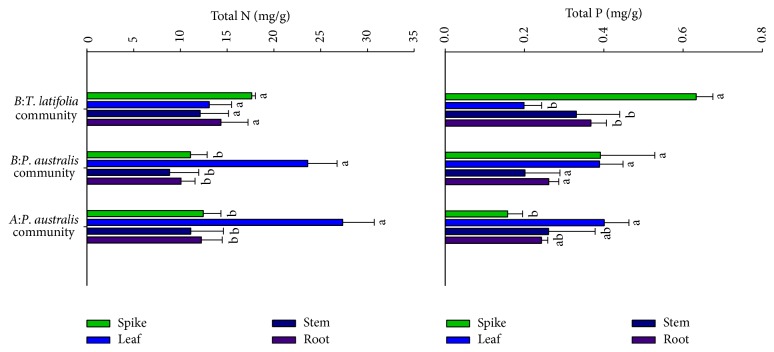
Concentration of TN and TP in the organs of* P*.* australis* and* T*.* latifolia* in experimental sites. Different lowercase letters indicate significant difference among TN and TP contents of organs (*P* < 0.05). Values are mean + 1 sem.

TN content was highest in* P*.* australis* leaves (*P* < 0.01), while little difference was observed between* P*.* australis* communities in sites A and B (*P* > 0.05, Figure 2). The total N content of* T*.* latifolia* leaves was lower than that of* P*.* australis* (*P* < 0.05), whereas other organs did not show significant difference in the total N content between the two species (*P* > 0.05). The total P content in both* P*.* australis* and* T*.* latifolia* varied significantly with the plant organ (Figure 2). It is 1.8- to 3.4-fold higher in* T*.* latifolia* spikes than in other organs. Conversely, TP content in the* P*.* australis* leaves was significantly higher than that of the spikes in the same community, which is consistent with the profile of TN allocation. Interestingly, TP was significantly increased in* P*.* australis* spikes in the* P*.* australis* +* T*.* latifolia* community, showing little difference to the leaves (*P* > 0.05). The P content in* P*.* australis* spikes of the mixed community is 2.5-fold higher than that of the* P*.* australis* community (*P* < 0.01).

The allocation of the nitrogen and phosphorus storage across organs in* P*.* australis* showed the same profile as roots > stems > leaves > spikes, while* T*.* latifolia* in the mixed community showed roots > spikes > stems > leaves (Table 2). The N and P storages in* P*.* australis* roots were 66.7% and 71.4% of the total N and P storage, which is higher than that in* T*.* latifolia* roots. By contrast, the N and P storages in spikes were significantly higher in* T*.* latifoli*a (*P* < 0.05). The N and P storages of* P*.* australis* in* P*.* australis* community were only 4.2% and 2.6% of the TN and TP storage. Nonetheless, TP storage of* P*.* australis* in* P*.* australis* +* T*.* latifolia* community was significantly increased, taking up to 7.2% of the total P storage. This is 2.8-fold higher than that in* P*.* australis* community. Due to the notable difference in plant biomass between* P*.* australis* and* T*.* latifolia*, the N and P storage were both high in* P*.* australis*. However, the high P content in* T*.* latifolia* spikes results in the total P storage in* T*.* latifoli*a community 2-fold higher than that in* P*.* australis* community.

### 3.4. The Seasonal Variations of TN and TP Contents in Plant Organs

**Figure 3:**
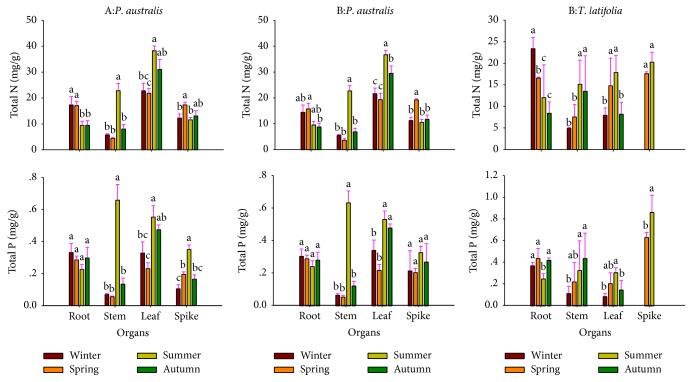
Seasonal dynamic concentration of TN and TP in the organs of* P*.* australis* and* T*.* latifolia* in experimental sites. The first and second columns of figure are the information of* P*.* australis *from sites A and B, and the third column is* T*.* latifolia *from site B. Different lowercase letters indicate significant difference among TN and TP contents of seasons (*P* < 0.05). Values are mean + 1 sem.

No data was collected on TN and TP content in* T*.* latifolia* spikes in late autumn and winter, during which the spikes disappear in response to the climate condition (Figure 3). The total N and P contents in* P*.* australis* stems and leaves were highest in summer, which is particularly true for TN. TN content in* P*.* australis* stems was higher in summer than other seasons up to 70–84%, while the P content up to 81–92%. TP content in* P*.* australis* roots did not vary with the season, which was likely due to the stable biomass and TP demands throughout the seasons, whereas, the high TP content in* P*.* australis* stems was likely due to the vigorous growth in summer that needs more TP than usual to promote plant growth. Similarly, TN contents in* T*.* latifolia* stems and leaves varied with season, both highest in summer, when the TN content among organs showed a pattern of spikes > leaves > stems > roots, while the P content was spikes > stems > leaves > roots. The high TN and TP contents in* T*.* latifolia* spikes were likely due to the fast growth and development in summer, which demands more TN and TP supply, compared to other seasons.

### 3.5. The Grey Correlation Analysis between Soil and the Contents of TN and TP about* P*.* australis* Organs

The association index of N content in* P*.* australis* and soil in different depth has negative correlation with soil depth, while the largest value of association index about P is the soil layer ≥15–30 cm (Table 3).* P*.* australis* needs N in the process of growth of different organs and obtains it mainly from the shallow soil, whereas the nutrient elements in the deep soil layer play a relatively small role in the growth of* P*.* australis*. There is no significant difference in various soil layers with the association index of P content, slightly increasing only in the soil layer ≥15–30 cm. It is in summer that the relationship of N and P contents is best in* P*.* australis* and soil. The plants thrive in summer, and the soil layer of 0–15 cm has large impact on* P*.* australis* gaining the N and P in summer which is a period of strong growth of plants. At the same time, as a result of the underground water level that rises higher in rain season, medium and lower soil layer may have greatly impact the content of nutrient elements in plants. This phenomenon is also reflected in spring, but the tendency is not significant compared with summer.

## 4. Discussions

Wetland soils play an important role in improving water quality, nutrient accumulation, and regeneration of nitrogen and phosphorus, which largely depends on the plant community structure [23, 24]. The notable reductions of TN and TP content in the downstream water of the research site indicated that Qin Lake wetland is able to remove the water N and P and that the ability of investigated communities to retain TN (reduced by 37.3%) is high as compared to TP (reduced by 27.3%), while* P. australis* and* T. latifolia* are two dominating aquatic plants, both of which are regularly used in construction wetlands to remove N and P from aquatic systems due to their competence in nutrient enrichment [25–27]. The enhanced N retention in* P*.* australis* soils, especially in the 30–45 cm soil layer, was mainly attributed to the roots whose 43% were distributed in the below 30 cm soils. Furthermore, 91% of them were fine roots less than 2 mm in diameter, but with a massive surface area and fast turnover rate, which dramatically enhances the N enrichment in below 30 cm soils.

In addition*, P*.* australis* +* T*.* latifolia* community significantly increased the wetland soil TN content, demonstrating that plant community structure has significant impact on the wetland soil N retention (Figure 1). It was likely due to the mechanism of interspecific adaptations, where* P*.* australis* adopted stress tolerance mechanism, while* T*.* latifolia* adopted stress escape mechanism to accommodate interspecific competition as they coexisted in the same environment [28]. The adaptation was reflected by a series of changes mainly in morphological phenotypes, such as the elongation of* T*.* latifolia* root, decrease in stem diameter, decrease of the leaf numbers, and the increase of plant height [29]. The competition between* P*.* australis* and* T*.* latifolia* improved the plant nitrogen use and uptake efficiencies and promoted the biological N enrichment process, which ultimately contributed to the increase of soil total N through the plant-soil system. Contrarily, the wetland soil P content was significantly decreased in the* P*.* australis* community and maintained the same concentration in the* P*.* australis* +* T*.* latifolia* community (Figure 1), which was mainly attributed to the strategy of these two species referring to the low P condition in the environment.* P*.* australis* is able to grow in low P condition by adjusting the rhizospheric soil pH to solubilize insoluble P and making it available for plant uptake, which expedites soil P consumption and, on the long run, depletes P in the surface soils [30]. By contrast,* T*.* latifolia* growing in the P deficient condition relies on increasing its root biomass to acquire P from deep soil solution to support their growth without changing the soil chemical compositions [31], which ultimately retains P through plant-soil system and offsets the soil P consumption by* P*.* australis* +* T*.* latifolia* community. The impact of* T*.* latifolia* on soil P retention is enhanced with the increase in soil depth. The P reduction in the 0–15 cm soil layers in the mixed community was primarily due to the* T*.* latifolia* roots distributed mainly in the deep soils, which may result from the escape mechanisms adopted during the interspecific competition [32, 33], which makes it unable to offset* P*.* australis* P consumption in the surface soils.

It had been reported that spike, as a reproductive organ, had comparatively higher N and P content than other organs, because of the high level of mitochondria content. However, the N and P content in* P*.* australis* spikes were not significantly higher than that of other organs in our study. It is likely due to the Qin Lake wetland eutrophication that changed the ecological strategy of* P*.* australis* community, where they reduced the cost of reproduction but increased the plant growth input. Similarly, the stem N and P contents were also low in both* P*.* australis* and* T*.* latifolia*, which is reasonable because it is mainly used to transport water and nutrients, as well as to provide plants physical support [34]. However, the total N and P contents in stem and leaves were highest in both species. This can be explained by the highest efficiency of photosynthesis and evapotranspiration in summer, when proteins, nucleic acids, and chloroplast are synthesized and transported more efficiently in these organs than any other seasons [12]. On the other hand, a large quantity of P is necessary to meet the need of energy consumed in the photosynthesis and respiration [35].

N : P ratio has been known as an indicator of N or P limitation [36]. N : P < 14 suggests N limits plant growth, while P becomes the limiting factor when N : P > 16 [37]. The N : P ratios in plant organs in this study are significantly different from the global and national N : P ratios for terrestrial plants. It is noteworthy that the average N : P ratio in leaves was 68.9, which was remarkably higher than that of the global (13) and national (14) averages. High leaf N : P ratio is beneficial to the photosynthesis and further promotes plant growth [38, 39]. The high leaf N : P ratio in this study is mainly due to the fact that the N content (26.87 g/kg) in* P*.* australis* leaves is well above the average of terrestrial plant leaves in China (18.6 g/kg) and that the P content (0.39 g/kg and 0.19 g/kg) in* P*.* australis* and* T*.* latifolia* leaves is, conversely, well below the average of terrestrial plant leaves in China (1.21 g/kg) [40]. The whole evidence indicated that the Qin Lake eutrophication is likely due to the excess of N, because P, relative to N, is still in deficiency, implying that P is the limiting factor of the aquatic plant growth in this region. It also suggested that* P*.* australis* community not only failed to improve the P retention efficiency, but also expedited the soil P consumption.

Nutrients exchange and enrichment occurred mainly in the surface soils. The root biomass of* P*.* australis* is mainly distributed in the surface soil (0–30 cm), while its vertical distribution could reach the 60 cm of soil layer. The content of N and P was often higher in the surface soil than that in deep soil due to the fresh soil solution supply from eutrophic runoff [41] and higher concentration of N, P, and organic matters [42], which led to intensive sequestration of nutrient elements in soil-vegetation system [43]. Meanwhile, the utilization efficiency of plant roots for N and P in deep soil was depressed by the lack of oxygen caused by the long-term water saturation [44].

According to the seasonal dynamic of correlation between N and P contents in* P*.* australis* and soil, the N and P sequestration of the wetland soil benefited from the growth of aquatic plants [45], especially in summer when the plants were at the time of most vigorous growth. The N and P in the runoff would be absorbed by plants, and then feedback to the soil as fine root litters and root exudates [46]. Therefore, the N and P contents in* P*.* australis* and the surface soil with the densest distribution of roots had the highest correlation.

## 5. Conclusion

*P*.* australis* +* T*.* latifolia* community enhanced the efficiency of N enrichment in wetland soil and was the same to the P comparing to* P*.* australis* community. Nutrient enrichment efficiency varies with the season and soil depth. The highest N and P enrichment efficiencies of the plant communities occurred in summer in 0–15 cm soil layer, while the enrichment of P occurred uniformly in all vertical soil layer.

## Figures and Tables

**Table 1:** Annual average concentrations of N and P in the water of upstream and downstream of experimental sites (*n* = 12).

TN (mg/L)			TP (mg/L)		
Upstream	Downstream	*P* value	Upstream	Downstream	*P* value
1.26 ± 0.20	0.79 ± 0.08	0.05	0.11 ± 0.01	0.08 ± 0.003	0.02

**Table 2:** Organs biomass and N and P reserves of *P. australis* and *T*.* latifolia *(*n* = 8).

	*P*.* australis *from plot A					*P*.* australis *from plot B					*T*.* latifolia *from Plot B				
	Root	Stem	Leaf	Spike	Total	Root	Stem	Leaf	Spike	Total	Root	Stem	Leaf	Spike	Total
Biomass (g/m^2^)	4512.0 ± 1308.1	1047.9 ± 991.70	343.5 ± 305.5	269.2 ± 229.4	6172.6 ± 1819.3	3965.5 ± 1822.6	1123.4 ± 887.02	389.8 ± 223.7	305.8 ± 189.1	5784.5 ± 2230.3	436.1 ± 110.0	75.8 ± 32.4	59.60 ± 15.70	124.2 ± 42.00	603.80 ± 152.90
N reserves (g/m^2^)	55.2	11.6	9.4	3.3	79.5	39.9	9.9	9.2	3.4	62.4	6.3	0.9	0.8	2.2	10.2
P reserves (g/m^2^)	1.1	0.3	0.1	0.04	1.54	1.0	0.2	0.1	0.1	1.4	0.2	0.03	0.01	0.08	0.32

Plot A is *P. australis* community; plot B is *P. australis* + *T*.* latifolia* community.

**Table 3:** Grey Correlation Analysis on contents of N and P between organs of *P. australis* and different depth of soil.

Depth of soil	Association index								Association index of N and P between the whole plant and different depth of soil	
	Winter		Spring		Summer		Autumn			
	N	P	N	P	N	P	N	P	N	P
0–15	0.30	0.40	0.30	0.54	0.51	0.72	0.25	0.25	0.71	0.65
≥15–30	0.35	0.40	0.25	0.45	0.45	0.66	0.35	0.25	0.68	0.66
≥30–45	0.43	0.36	0.25	0.45	0.52	0.64	0.33	0.25	0.67	0.65
≥45–60	0.41	0.33	0.26	0.62	0.41	0.70	0.25	0.25	0.64	0.65
Seasonal association index of N and P between the whole plant and soil	0.37	0.37	0.27	0.52	0.47	0.68	0.30	0.25		
